# Infections as a cause of autoimmune rheumatic diseases

**DOI:** 10.1007/s13317-016-0086-x

**Published:** 2016-09-14

**Authors:** Lazaros I. Sakkas, Dimitrios P. Bogdanos

**Affiliations:** Department of Rheumatology and Clinical Immunology, University of Thessaly Medical School, Biopolis, 40 500 Larissa, Greece

**Keywords:** Autoimmunity, Infection, Rheumatic disease, Rheumatoid arthritis, Systemic sclerosis

## Abstract

Exogenous and endogenous environmental exposures and particularly infections may participate in the breakage of tolerance and the induction of autoimmunity in rheumatic diseases. Response to infections apparently occurs years before clinical manifestations and features of autoimmunity, such as autoantibodies, are detected years before clinical manifestations in autoimmune rheumatic diseases. In this review, we summarize the current evidence for a potential causal link between infectious agents and rheumatoid arthritis, systemic lupus erythematosus, systemic sclerosis, Sjogren’s syndrome and ANCA-associated vasculitis.

## Introduction

Infectious agents have long been suspected as initiating agents (etiology) of rheumatic diseases. In the 19th century, the belief that rheumatoid arthritis (RA) was caused by mycobacteria led to treatment of rheumatoid arthritis with gold salts used for the treatment of infectious diseases. Epidemiological and family studies have shown that environmental factors play a significant role in the development of rheumatic diseases [1]. This is exemplified by the low concordance rate of RA in monozygotic twins but higher than that in dizygotic twins. Moreover, environmental factors appear to work in a proper genetic background in various autoimmune rheumatic diseases [2]. Infectious agents are part of the environmental insults to human beings. Infectious agents can cause autoimmunity and autoimmune disease by various mechanisms. For instance, an immune response to an infectious agent may result in an autoimmune disease by molecular mimicry, epitope spreading, bystander activation or pathogen persistence [3, 4]. Another mechanism is through epigenetic changes [5, 6]. Bacterial agents but also commensal bacteria can cause epigenetic modification of host genes. Epigenetic changes are DNA modification without change in nucleotide sequence and post-translational histone modification, all of which change chromatin configuration and thus accessibility of genes to transcription machinery. For example, intestinal commensal bacteria affect DNA methylation of the Toll-like receptor 4 (TLR4) gene of the host that recognizes the lipopolysaccharide of Gram (−) bacteria [7]. Another means of epigenetic modification is through microRNAs (miRNAs). miRNA is a small (20–30 nucleotide long) non-coding RNA that silences the target gene by binding to its mRNA [8]. Besides endogenous miRNAs, exogenous miRNAs can affect the expression of human genes. For example, miR168a from consumed rice can bind to human and mouse LDL receptor protein-1 mRNA and inhibit its translation [9].

In the following sections, we will present epidemiological, clinical, immunological and experimental data that link autoimmune rheumatic diseases with specific infectious agents.

### Rheumatoid arthritis

Rheumatoid arthritis is a chronic inflammatory polyarthritis that affects most commonly the small joints of the hands and feet and may affect extra-articular tissues and organs, most importantly lungs and the cardiovascular system. In RA, environmental factors appear to play a more significant role than genetic factors. The concordance rate of RA around 14 % in monozygotic twins and 4 % in dizygotic twins suggests a rather small influence of genetic factors on the development of the disease [10–13]. Two environmental factors are known as risk factors for RA, namely periodontitis and cigarette smoking [10, 11, 14–16]. Among genetic factors, HLA genes are the best studied genes in RA. RA is associated with HLA-DRB1* alleles carrying a common amino acid sequence at position 70–74 of the β chain, which is refered to as shared epitope (SE, HLA-DRB1*SE) [17, 18]. HLA-DRB1* alleles on antigen-presenting cells present antigen to T cells. Therefore, and given that interferon (IFN)-γ (a Th1 product) and interleukin(IL)-17 (a Th17 product) are elevated in RA, the association with the HLA-DRB1*SE suggests that in RA, HLA-DRB1*SE alleles present an arthritogenic peptide to T cells to initiate an immune response that culminates in a cytokine cascade with IFN-γ, IL-17, tumor necrosis factor (TNF)-α and IL-6 [19, 20]. Alternatively, the HLA-DRB1*SE itself may be the target of an immune response. For instance, the Epstein-Barr virus (EBV) gp110 glycoprotein shares sequence homology with HLA-DRB1*SE and an initial immune response to EBV may later also involve human HLA-DRB1*SE by molecular mimicry [21].

For many years, rheumatoid factor was the only evidence for autoimmunity in RA. In recent years, citrullinated proteins have been shown to be the targets of B cells and T cells in RA. Citrulline derives from arginine residues by post-translational modification of proteins through the action of the enzyme peptidylarginine deiminase (PAD). Anti-citrullinated peptide antibodies (ACPAs) appear up to 10 years before the onset of clinical arthritis in RA [22, 23] and are a strong susceptibility factor for RA [23–25]. In fact, ACPAs are detected in around 70 % of patients with RA, and are correlated with the severity of the disease [26, 27]. More interestingly, ACPAs are associated with HLA-DRB1*SE [23–25]. The apparent explanation for association is that T cells recognize citrullinated peptides sitting on HLA-DRB1*SE on B cells and provide help to B cells for the production of ACPAs. Indeed, HLA-DRB1*SE alleles bind to citrullinated peptides in RA, as citrulline but not arginine was eluted from HLA-DRB1*04:01/04(SE) alleles [28]. In addition, CD4(+) T cells from the peripheral blood of HLA-DRB1*04:01 (an HLA-DRB1*SE allele) patients with RA, were found to recognize citrullinated vimentin and citrullinated aggrecan [28]. Furthermore, oligoclonal expansions of T cells were detected in synovial biopsies from ACPA(+) RA patients compared to ACPA(−) RA patients [29, 30]. It is worth reminding that oligoclonal expansion of T cells indicates an antigen-driven activation and proliferation of T cells.

As mentioned, two environmental factors, namely periodontitis and cigarette smoking, are risk factors for RA and may exert this susceptibility via protein citrullination and ACPA production. Cigarette smoking is a strong inducer of protein citrullination in a proper genetic background. Furthermore, cigarette smoking is a risk factor for ACPA in RA patients carrying the HLA-DRB1*SE [31], and this tobacco exposure-HLA-DRB1*SE interaction has been confirmed in a number of studies [32–34]. Animal models provide explanation for this association: tobacco exposure induces PAD in transgenic mice carrying RA-susceptible HLA-DR alleles [35], thus providing a means for new antigens (autoantigens) to the immune system. *P. gingivalis*, a microbe that is the major causative agent for periodontitis, possesses PAD that can cause citrullination of both bacterial and host proteins [36]. A citrullinated α-enolase peptide-1 (CEP-1) was identified as a dominant B cell epitope present in 36–60 % of RA patients [37]. It is worth mentioning that CEP-1 is highly conserved in prokaryotes and eukaryotes, and human CEP-1 shares 100 % homology of a 9 amino acid span with *P. gingivalis* α-enolase [37]. Antibodies to human CEP-1 cross-reacted with recombinant *P. gingivalis* α-enolase [37] and anti-citrullinated bacterial α-enolase antibodies are detected in ACPA(+) RA patients [38]. *P. gingivalis* can contribute to RA through another mechanism. *P. gingivalis* DNA was detected in synovial fluid from RA patients more frequently than in controls (15.7 vs 3.5 %) [39]. Furthermore, *P. gingivalis* DNA can induce IL-1, IL-6 and TNFα production in a monocytic cell line through TLR9 [40]. These findings suggest that bacterial persistence in the joints may also contribute to the synovial inflammation in RA.

Active EBV infection also appears to contribute to synovial membrane (SM) expansion and differentiation of autoreactive B cells. For instance, in ectopic lymphoid, follicle-like structures (ELS)-containing RA synovial membrane, latent and lytic EBV infection were detected, and a large proportion of plasma cells producing ACPAs were infected with EBV. Furthermore, ELS-containing RA SM transplanted into severe combined immunodeficiency (SCID) mice produced ACPAs and anti-EBV antibodies [41]. All the above data point to the notion that cross-reactivity between bacteria and human citrullinated proteins can break tolerance and induce arthritis.

The finding of an autoantigen does not prove its pathogenicity, i.e., cause of tissue injury. Experimental data support the notion that citrullinated peptides are arthritogenic autoantigens in RA. Thus, both citrullination of proteins and the HLA-DRB1* SE, are required for the development of arthritis: citrullinated fibrinogen but not unmodified fibrinogen could induce arthritis in transgenic mice carrying DRB1*04:01 (an HLADRB1*SE allele). On the other hand, citrullinated or unmodified fibrinogen could not induce arthritis in wild-type (B6) mice [42]. ACPAs against citrullinated vimentin induce osteoclastogenesis and bone loss, cardinal features of joint involvement in RA [43]. Also immune complexes containing citrullinated fibrinogen stimulated macrophage TNFα production through TLR4 and Fcγ receptor [44]. In collagen-induced arthritis, a PAD inhibitor reduced the severity of arthritis, an effect that supports an arthritogenic role for citrullination and ACPA production in RA [45]. Furthermore, *P. gingivalis* infection exacerbated collagen-induced arthritis (CIA), and this exacerbation was dependent on the expression of *P. gingivalis* PAD [46].

Citrullinated antigens are detected in neutrophil extracellular traps (NETs), formed spontaneously or in stimulated RA neutrophils [47, 48]. NETs are structures of decondensed chromatin and granule antimicrobial lysosomal proteins, such as proteinase-3, myeloperoxidase, lactoferrin, elastase and others. NETs are extruded from neutrophils while dying (NETosis) to kill bacteria [49].

ACPAs may be produced in lymphoid organs, as most antibodies, or in local tissues. Higher expression of PAD2 was detected in bronchial mucosa and bronchoalveolar lavage cells in healthy smokers compared to non-smokers [50]. The inflamed synovial membrane of RA is a site for ACPA production, since ACPA levels were higher in synovial fluid compared with serum from the same patients [24, 51]. Further supporting evidence comes from the finding that the majority of synovial membrane IgG-expressing B cells are specific for citrullinated autoantigens in ACPA(+) RA patients [52]. It has already been mentioned that ACPAs are produced in RA synovial membrane as ELS-containing RA SM transplanted into SCID mice produced ACPAs along with anti-EBV antibodies [41].

The gut microbiome may also affect the immune response in a proper genetic background in RA. For example, transgenic mice carrying the RA-susceptible allele HLA-DRB1*04:01 have a differential Th17 cytokine profile and do not exhibit the sex- and age-difference in gut microbiome that transgenic mice carrying the RA-resistant allele HLA-DRB1*04;02 exhibit [53].

### Systemic sclerosis

Systemic sclerosis (SSc) is a chronic systemic disease characterized by fibrosis of the skin and internal organs, vasculopathy, and activation of the immune system. Vasculopathy comprises of vasospastic episodes (Raynaud’s phenomenon, RP) and fibrointimal proliferation of small vessels, whereas immune activation is evident by serum autoantibodies detected in patients with SSc, and the oligoclonal expansion of T cells in skin lesions [54]. The best known autoantibodies in SSc are antinuclear antibodies and anti-topoisomerase I antibodies (formerly Scl70), which are associated with diffuse cutaneous disease, and anti-centromere antibodies, which are associated with limited cutaneous disease. RP and autoantibodies appear years before clinical manifestations of fibrosis, and microvascular damage (as detected by nailfold capillaroscopy) and autoantibodies are independent predictors for the progression of RP to SSc [55]. The pathogenesis of SSc is incompletely understood [56]. In the avian scleroderma model, endothelial cell apoptosis was the earliest change detected [57]. Environmental factors play a major role in the development of the disease since the concordance rate of SSc in monozygotic twins is low (4.7 %) and equal to dizygotic twins [58]. Molecular mimicry has been suggested as early pathogenetic mechanism for SSc and several microbes have been implicated, including human cytomegalovirus (hCMV), EBV, endogenous retroviruses and *H. pylori.* The strongest data supporting a pathogenetic role in SSc holds for hCMV and EBV. Early studies reported increased serum levels of anti-hCMV antibodies in SSc patients [59]. In addition, SSc patients have antibodies against an epitope of the hCMV late protein UL94, that shares homology with the novel antigen-2 (NAG-2), present on endothelial cells. Anti-UL94 antibodies bind to NAG-2 on endothelial cells and induce apoptosis [60]. NAG-2 is also expressed on human fibroblasts and anti-UL94 antibodies bind to fibroblasts that acquire a profibrotic phenotype [61]. Furthermore, hCMV-derived UL70 protein shares homology with Topoisomerase I. hCMV is also associated with increased risk of graft-versus-host disease (GVHD), a condition that develops after bone marrow transplantation, shares clinical and serological features with SSc and is considered a model for SSc [62]. Murine CMV (mCMV) can invade endothelial cells in mice and cause latency and intermittent shedding of the virus. mCMV-infected irradiated interferon-γ receptor knock-out (IFNγR−/−) mice exhibit neointima formation with myofibroblast proliferation in small vessels [63].

EBV is another candidate causative agent for SSc. EBV is a lymphotropic virus infecting the vast majority of adult population. EBV causes latency but is also reactivated into lytic infection and, besides B cells, can infect the majority of fibroblasts and endothelial cells in the skin of patients with SSc. Furthermore, EBV activates fibroblasts towards profibrotic phenotype through TLR, TGFβ1 and endothelin [64]. Parvovirus B19 may also participate in SSc pathogenesis, since parvovirus B19 DNA was detected in the bone marrow of SSc patients but not in controls [65].

Inflammasome, activated by dangerous stimuli and through the action of caspase, induces the production of inflammatory mediators, such as interleukin-1, and is activated in SSc. Increased expression of NLRP3 and AIM2 inflammasome proteins was detected in SSc skin fibroblasts, while inhibition of caspace abrogated the secretion of collagen, IL-1β and IL-18 [66]. It should be mentioned that the AIM2 inflammasome is a sensor for cytosolic bacterial and viral DNA [67].

### Systemic lupus erythematosus

Systemic lupus erythematosus (SLE) is a multisystem disease affecting mostly women in reproductive years. It is characterized by many autoantibodies [68], including antinuclear antibodies, anti-dsDNA antibodies, anti-Sm antibodies and anti-Ro antibodies. Both genetic and environmental factors interplay for the development of the disease [69] as the concordance rate of SLE in monozygotic twins (24 %) is higher than that in dizygotic twins (2 %) [70]. EBV has long been suspected to play a pathogenic role in SLE. EBV-IgA antibodies, which are thought to reflect reactivation or re-infection with EBV, were associated with SLE, particularly in African-Americans [71, 72]. Antibodies to EBV nuclear antigen-1 (EBNA-1) and EBNA-2 cross-react with SmD and 60 kD Ro, and mice or rabbits immunized with EBNA-1 develop experimental lupus [73, 74]. It should be mentioned that 44 % of patients with primary acute EBV infection have serum antibodies against extractable nuclear antigens (ENA) [75].

Retroviruses are also candidate agents in SLE [76]. Retroviruses are small viruses that use reverse transcription for their replication. Human endogenous retroviruses (HERV) are retroviruses thought to be trapped into the human genome. These retroviruses can be activated by many environmental factors, such as infections, ultraviolet (UV) light, hormones, stress and drugs [76]. In EBV latency infected B cells, there is transactivation of HERV-K18 that codes for the *env* protein, a T cell superantigen. T cell superantigens bind to Vβ segment of T cell receptor and activate a huge proportion of T cells. Another HERV, HERV3, codes for an *env* protein expressed in placenta and shares homology with the Ro antigen. For long it has been known that mothers with anti-Ro antibodies have increased risk for fetal heart block (congenital heart block, CHB) and mothers of babies with CHB have anti-HERV3 antibodies that bind to sections of fetal heart [77].

Epigenetic changes caused by infections may also be another pathogenetic mechanism operating in SLE. Environmental factors, such as infection, drugs, smoking and UV light, cause oxidative stress and DNA demethylation of certain genes, such as genes of CD4+ T cells to become autoreactive cells [78]. CD4+ T cells treated with a DNA methylation inhibitor (5-azacytidine, 5-azaC) overexpress CD11a, perforin, CD40L (costimulatory molecule), CD70 (B cell costimulatory molecule), killer cell immunoglobulin-like receptor (KIR, not normally expressed on T cells) and stimulate autologous B cells. Similarly, CD4+ T cells from SLE patients overexpress CD11a, perforin (not normally expressed in T cells), CD40L, CD70 and KIR [76, 78].

### Sjögren’s syndrome

Sjögren’s syndrome (SS) is a chronic autoimmune disease, more prevalent in women, affecting exocrine glads, mostly salivary and lacrimal glands, but also extraglandular tissues and organs. SS is characterized by relatively specific autoantibodies, namely anti-Ro (SSA), anti-La (SSB), and by ELS in exocrine glands. Hepatitis C virus (HCV), EBV and human T cell leukemia virus (HTLV)1 have been put forward as causative agents in SS. In a meta-analysis, SS has been associated with HCV [79]. Active EBV infection appears to cause expansion and differentiation of autoreactive B cells in SS. Latent EBV and lytic EBV infection was detected in ELS-containing SS salivary glands and plasma cells with Ro52 immunoreactivity were frequently infected by EBV. Furthermore, ELS-containing SS salivary glands transplanted into SCID mice produced anti-Ro52 antibodies and anti-EBV antibodies [41]. Commensal microbiota may initiate autoimmunity in SS and SLE. For instance, peptides from the von Willebrand factor type A from the oral microbe *Capnocytophaga ochracea* activated HLADR3 (+), Ro60-reactive T cells [80]. Environmental pollutants, such as dioxin, through aryl hydrocarbon receptor, reactivates (switches from latent to lytic infection) EBV in B cells and salivary epithelial cells [81]. HTLV1 is associated with SS in endemic areas, such as Nagasaki in Japan [82, 83]. It should be mentioned that HTLV1 preferentially transfects CD4 + T cells, but can also transfect human primary salivary gland epithelial cells [82].

### Vasculitis

Vasculitis is idiopathic inflammation of vessel wall. There are various types of vasculitis classified according to vessel size preferentially involved.

#### ANCA vasculitis

Vasculitis associated with anti-neutrophil cytoplasmic antibodies (ANCA vasculitis) encompasses granulomatosis with polyangiitis (GPA, formely Wegener’s granulomatosis), eosinophilic granulomatosis with polyangiitis (EGPA, formely Churg-Strauss syndrome) microscopic polyangiitis, and pauci-immune glomerulonephritis (focal necrotizing glomerulonephritis, FNGN). The characteristic features of these vasculitides are the presence of ANCA in the sera of patients and the absence of immune deposits in the glomeruli on immunofluorence in patients with glomerulonephritis (pauci-immune GN). The mechanisms responsible for the induction of these diseases are poorly understood. Classical ANCA’s target is the antimicrobial lysosomal enzyme either proteinase-3 or myeloperoxidase [84]. A long standing clinical observation of increased frequency of nasal carriage of *S.aureus* in patients with GPA has linked ANCA vasculitis with infectious agents [85]. This observation has led to antimicrobial treatment of GPA with beneficial effects. Antibodies against complementary proteinase-3 (cPR3) were found in GPA and cPR3 has homology with *S. aureus* antigens [86]. A new and somewhat controversial ANCA subtype, namely anti-lysosomal membrane protein-2 (LAMP-2), has been linked to ANCA-associated vasculitis. Patients with FNGN have antibodies to LAMP-2 epitope 41-49 that has 100 % homology with FimH, an adhesion molecule present on Gram(−) bacteria whereas immunization with FimH-induced anti-LAMP-2 antibodies and FNGN [87]. Thus, FNGN provides a direct link for a molecular mimicry between bacteria and host proteins. As found in RA, ANCA vasculitis is associated with increased formation of NETs. NETs can provide autoantigens to dendritic cells and activate B cells [88]. *S. aureus* and ANCAs are strong inducers of NET formation [89].

#### Other vasculitides

Other types of vasculitides are also associated with infectious agents. Mixed cryoglobulinaemic vasculitis is associated with HCV. In fact, 70–100 % of patients with mixed cryoglobulinaemic vasculitis have evidence of HCV infection, hence the term HCV-related mixed cryoglobulinaemia. HCV is a RNA virus and causes chronic infection and hence persistent antigenic stimulus that leads to monoclonal IgM rheumatoid factor production, immune complex formation and complement activation [90].

Henoch-Schonlein purpura, a small vessel vasculitis, primarily in children, has been associated with group A streptococci, parvovirus B19 and others infectious agents. Kawasaki disease, which affects medium-sized arteries, has been associated with viral agents [91], and polyarteritis nodosa is associated with hepatitis B virus [92].

## Conclusion

Interaction between genes and environmental factors, particularly infectious agents appear to be involved in the development of autoimmune rheumatic diseases. Thus far, cigarette smoking and infectious agents causing periodontitis are clearly two environmental agents with the strongest evidence for interaction with genes (HLA-DRB1*SE) in the pathogenesis of RA. The definitive identification of infectious agents implicated in other autoimmune rheumatic diseases requires further investigations.

## Abbreviations

ab: Antibody
ACPA: Anti-citrullinated peptide antibody
CEP-1: Citrullinated α-enolase peptide-1
CIA: Collagen-induced arthritis
EBV: Epstein-Barr virus
EBNA-1: EBV nuclear antigen-1
ELS: Ectopic lymphoid follicle-like structures
GVHD: Graft-versus-host disease
hCMV: Human cytomegalovirus
HCV: Hepatitis C virus
HTLV: Human T cell leukemia virus
IFN: Interferon
IL: Interleukin
LAMP: Lysosomal membrane protein-2
mCMV: Murine cytomegalovirus
NET: Neutrophil extracellular traps
PAD: Peptidylarginine deiminase
RA: Rheumatoid arthritis
SM: Synovial membrane
SS: Sjögren syndrome
SSc: Systemic sclerosis
TLR: Toll-like receptor
TNF: Tumor necrosis factor
